# Multiple molecular dynamics simulation of the isoforms of human translation elongation factor 1A reveals reversible fluctuations between "open" and "closed" conformations and suggests specific for eEF1A1 affinity for Ca^2+^-calmodulin

**DOI:** 10.1186/1472-6807-8-4

**Published:** 2008-01-25

**Authors:** Dmitry S Kanibolotsky, Oleksandra V Novosyl'na, Catherine M Abbott, Boris S Negrutskii, Anna V El'skaya

**Affiliations:** 1 Institute of Molecular Biology and Genetics, National Academy of Sciences of Ukraine, 150 Academician Zabolotny Street, 03680 Kiev, Ukraine; 2 National Taras Shevchenko University of Kiev, 64 Volodymyrska Street, 01033 Kiev, Ukraine; 3 Medical Genetics, Molecular Medicine Centre, University of Edinburgh, Western General Hospital, Edinburgh EH4 2XU, UK

## Abstract

**Background:**

Eukaryotic translation elongation factor eEF1A directs the correct aminoacyl-tRNA to ribosomal A-site. In addition, eEF1A is involved in carcinogenesis and apoptosis and can interact with large number of non-translational ligands.

There are two isoforms of eEF1A, which are 98% similar. Despite the strong similarity, the isoforms differ in some properties. Importantly, the appearance of eEF1A2 in tissues in which the variant is not normally expressed can be coupled to cancer development.

We reasoned that the background for the functional difference of eEF1A1 and eEF1A2 might lie in changes of dynamics of the isoforms.

**Results:**

It has been determined by multiple MD simulation that eEF1A1 shows increased reciprocal flexibility of structural domains I and II and less average distance between the domains, while increased non-correlated diffusive atom motions within protein domains characterize eEF1A2. The divergence in the dynamic properties of eEF1A1 and eEF1A2 is caused by interactions of amino acid residues that differ between the two variants with neighboring residues and water environment.

The main correlated motion of both protein isoforms is the change in proximity of domains I and II which can lead to disappearance of the gap between the domains and transition of the protein into a "closed" conformation. Such a transition is reversible and the protein can adopt an "open" conformation again. This finding is in line with our earlier experimental observation that the transition between "open" and "closed" conformations of eEF1A could be essential for binding of tRNA and/or other biological ligands.

The putative calmodulin-binding region Asn311-Gly327 is less flexible in eEF1A1 implying its increased affinity for calmodulin. The ability of eEF1A1 rather than eEF1A2 to interact with Ca2+/calmodulin is shown experimentally in an ELISA-based test.

**Conclusion:**

We have found that reversible transitions between "open" and "close" conformations of eEF1A provide a molecular background for the earlier observation that the eEF1A molecule is able to change the shape upon interaction with tRNA. The ability of eEF1A1 rather than eEF1A2 to interact with calmodulin is predicted by MD analysis and showed experimentally. The differential ability of the eEF1A isoforms to interact with signaling molecules discovered in this study could be associated with cancer-related properties of eEF1A2.

## Background

**Figure 1:**
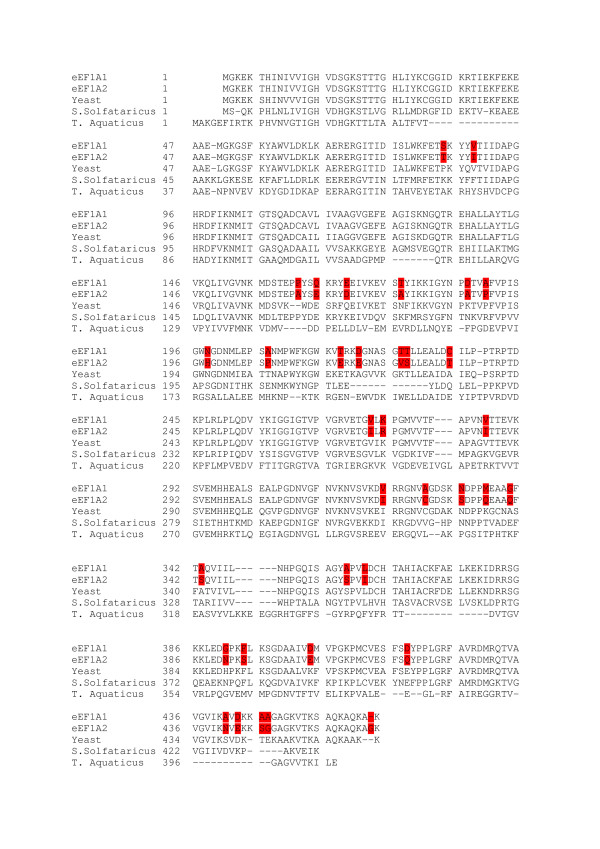
**Alignment of the sequences of elongation factors 1A**. eEF1A1 – human eEF1A1 [Swiss-Prot: P68104], eEF1A2 – human eEF1A2 [Swiss-Prot: Q05639], Yeast – yeast eEF1A [Swiss-Prot: P02994], S. Solfataricus – aEF1A of Sulfolobus solfataricus [Swiss-Prot: P35021], T. Aquaticus – EF-Tu of Termus Aquaticus [Swiss-Prot: Q01698]. Replacements and deletion in eEF1A1 and eEF1A2 are marked in red.

Higher eukaryotic translation elongation factor eEF1A operates in translation cycles by directing the correct aminoacyl-tRNA to the A site of mRNA-programmed ribosome [1]. A further translational function could be the interaction of eEF1A with deacylated tRNA coupled with direct transfer of tRNA in the channeled elongation steps of mammalian translation [2]. Besides its role in translation, eEF1A is involved in other cellular processes such as carcinogenesis and apoptosis and can interact with a large number of non-translational ligands in the cell [3,4]. There are two tissue and developmental-specific isoforms of eEF1A, which are 93% identical and 98% similar [5]. Importantly, despite the strong similarity of sequence at the amino acid level, the isoforms appear to differ in some properties and functions, which are both related and unrelated to translation. For example, the eEF1A2 isoform has a higher affinity for GDP than for GTP, whilst the affinity of eEF1A1 for these ligands is similar [5]. In the presence of eEF1A2 an inhibition of apoptotic processes was observed, whilst the opposite was found for eEF1A1 [6]. Importantly, the appearance of eEF1A2 in tissues in which the variant is not normally expressed can be coupled to cancer development, as shown for ovary and suggested in some cases of breast cancer [7-9]. Importantly, eEF1A1 serves as a housekeeping protein in the same tissues and seemingly is not related to the cancer in these cases. The structural peculiarities which underlay the cancer specificity of eEF1A2 remain unknown. The eEF1A1 and eEF1A2 isoforms contain 462 and 463 amino acid residues respectively with 34 replacements and a sole deletion of the penultimate residue in eEF1A1 (Figure 1). eEF1A1 has seven modified residues: N-trimethyllysines (M3l) 36, 79 and 318, N-dimethyllysines (Mly) 55 and 165, L-glutamyl 5-glycerylphosphorylethanolamines (GPE) 301 and 374 [10]. The eEF1A2 molecule is characterized by four modified residues (M3l55 and 165, GPE301 and 374), however, complete analysis of the eEF1A2 post-translation modifications is lacking [5]. Since the functional difference of the isoforms cannot be attributed to the any amino acid substitutions at our current level of knowledge, we reasoned that the background for the functional difference of eEF1A1 and eEF1A2 might lie in changes of spatial structure of the proteins.

Higher eukaryotic eEF1A has so far been resistant to attempts to crystallize it, so X-ray analysis is available only for the complex of yeast eEF1A with the truncated GDP exchange subunit eEF1Ba [11,12]. The identity of yeast and human eEF1A is more than 80%, so we modeled the spatial structures of human eEF1A1 and eEF1A2 using this X-ray structure as the main template. Not surprisingly, no meaningful difference in the static structures of eEF1A1 [see Additional file 1] and eEF1A2 [see Additional file 2] was found by this approach. In spite of this, it was tempting to analyze the molecular dynamics (MD) of the isoforms. In this case, initial models similar for both isoforms could be developed in the course of MD simulation into noticeably different conformations of the eEF1A1 and eEF1A2 molecules.

Complete conformational sampling for a three-domain protein may require an MD trajectory of a relatively long time scale. However, the protein can adopt denatured forms during simulation, and if the only trajectory is available, the denatured state can be interpreted as the native conformation in solution. Moreover, statistical errors accumulate at long MD calculation. In view of the aforesaid, an alternative, multiple MD simulation method [13,14] has been used. The method consists of the simulation of several relatively short MD trajectories starting from the same initial protein conformation with different initial atom velocities. Multiple MD simulation permits a reduction of computational time, a widening of the statistical basis, and allows us to evaluate quality of single trajectories and minimize force-field induced artifacts [15,16].

Our multiple MD simulation studies demonstrated more inter-domain mobility of the eEF1A1 molecule when compared to eEF1A2. At the same time eEF1A2 was characterized by a higher internal mobility of the structural domains. Amino acid residues were determined, for which flexibility is evidently different in the isoforms. Some significant MD characteristics inherent to both isoforms were also revealed; in particular, fluctuation of the eEF1A molecule between "open" and "closed" conformations in solution was shown providing for the first time a description of the dynamic behavior of human eEF1A in solution. Importantly, a difference in putative calmodulin binding sites of the isoforms has been predicted. The data obtained are an essential step in the move towards an understanding of the functional divergence of the near-identical eEF1A1 and eEF1A2 isoforms, and in particular, the cancer-related properties of the latter.

## Results and Discussion

**Figure 2:**
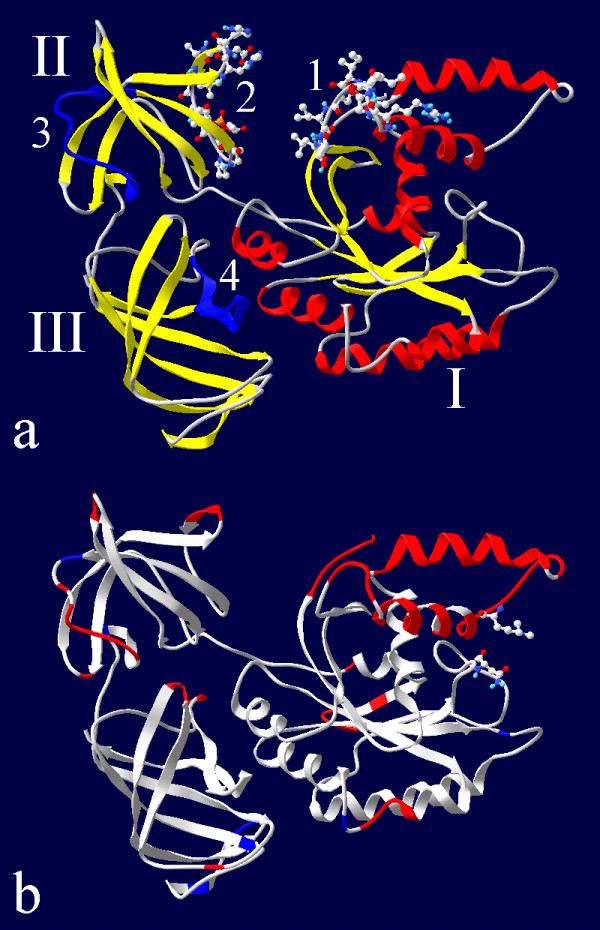
**Ribbon representation of the eEF1A molecule**. a – domains I, II and III; 1 and 2 – amino acid residues Arg69-Leu77 and His295-Gly305, which are on the surface of the gap between the domains I and II, 3 and 4 – motifs Asn311-Gly327 and Gly422-Val437, suggested to be the calmodulin binding site in eEF1A1. b – amino acid residues with a positive difference between the rmsf of eEF1A2 and eEF1A1 of more than 0.02 nm (Met1-Lys5, Asp35-Glu48, Gly50-Thr58, Asp61, Lys62, Lys64-Glu68, Gly70-Asp74, Ala125-Ser128, Val216-Gly221, Ala223, Cys/Thr234, Gly258, Ile259, Pro282, His296-Ala298, Ser316, M3l318-Arg322, Gln352, His364, Thr365, Tyr418-Pro420, ribbon colored in red) and with negative differences of less than -0.02 nm (Asn130, Ser157, Lys313, Ser329, Ser383, Gly384, Glu388, Asp398, ribbon colored in blue); side chains of residues Asp197 and Mly55 which stabilize the motif Asp35-Asp74 in eEF1A1 are shown.

The models consist of three domains (Figure 2a). The first domain contains 8 *β*-strands (Thr6-Ile13, Trp78-Thr82, Tyr85-Ala92, Cys111-Ala118, Gln147-Asn153, Ala189-Ile193, Trp214-Arg218 and Gly221-Gly225), which form the *β*-sheet surrounded by 8 *α*-helices (Lys20-Lys30, M3l/Lys36-Glu48, Ala57-Glu68, Asp97-Thr104, Val120-Ala125, Thr133-Leu143, Gln164-Ile181 and Leu228-Asp233). The second domain is the *β*-barrel formed by the strands Leu248-Leu250, Asp252-Ile256, Gly260-Val267, Met276-Ala281, Val/Ile285-Val289, Ser291-Met294, Glu297-Leu299, Asp306-Val312 and Asn324-Ser329. The third domain is also in the *β*-barrel consisting of *β*-strands Gly/Gln339-Leu347, Tyr357-Cys363, Ala366-Asp380, Lys385-Gly/Asn390, Asp398-Gly407, Gly422-Asp428 and Gln431-Asp/Glu442. In the present study the residues up to Pro238 are attributed to domain I, because Pro238 is the last residue from the continuous series of residues in the unstructured chain Cys234-Arg247, which are situated within the distance of the van der Waals radii sum to a residue from the other chain of the domain I (Asp110 for Pro238). Similarly, Pro241 is the first residue from the chain Cys234-Arg247, which is within the distance of the van der Waals radii sum from a residue of other chain of the domain II (Gly270), so Pro241 is chosen as the first residue of the domain II. Ser329 is the end of the domain II because it is the last residue in the Asn324-Ser329 *β*-strand. Domain III starts with Met/Gln335, which is situated within the distance of the van der Waals radii sum from a residue of other chain of the domain III (Cys411). Domains I and III and also domains II and III are situated tightly one to another, while a voluminous space exists between domains I and II. The space is limited by loop Arg69-Leu77 from the side of domain I and by residues His295-Gly305 from the side of domain II.

Table 1 demonstrates that the average rmsd of eEF1A1 is somewhat larger than rmsd of eEF1A2, consequently, the final solution conformation of eEF1A1 moves further away from the conformation of the initial model. Besides, since eEF1A1 rmsd is characterized by a larger *σ *value than rmsd of eEF1A2, the eEF1A1 molecule should have more conformational space than eEF1A2.

**Table 1:** Average values and root-mean-square fluctuations of parameters calculated from MD trajectories after 4000 ps

Parameter	eEF1A1		eEF1A2	
	
	M ± 2 m	*σ*	M ± 2 m	*σ*
C_*α*_-atoms trace rmsd for full protein	0.36540 ± 0.00058	0.05404	0.35817 ± 0.00045	0.04326
C_*α*_-atoms trace rmsd for domain I	0.30890 ± 0.00026	0.02438	0.31012 ± 0.00040	0.03861
C_*α*_-atoms trace rmsd for domain II	0.18125 ± 0.00030	0.02758	0.18347 ± 0.00042	0.04051
C_*α*_-atoms trace rmsd for domain III	0.17547 ± 0.00014	0.01277	0.20157 ± 0.00024	0.02270
Distance between centers of the domains I and II for all trajectories	3.26007 ± 0.00193	0.17880	3.33949 ± 0.00196	0.18727
Distance between centers of the domains I and II excluding trajectories 1,6,7,8,13 with "close" conformation for more than 500 ps	3.33688 ± 0.00159	0.11779	3.44489 ± 0.00212	0.14451
Distance between centers of the domains I and III	3.06352 ± 0.00087	0.08099	3.04624 ± 0.00069	0.06581
Distance between centers of the domains II and III	2.72471 ± 0.00050	0.04679	2.70452 ± 0.00048	0.04580
C_*α*_-atoms trace rmsd of Asn311-Gly327 after fitting of domain II to the initial conformation	0.18791 ± 0.00025	0.02324	0.18904 ± 0.00045	0.04320
C_*α*_-atoms trace rmsd of Gly422-Val437 after fitting of domain III to the initial conformation	0.17349 ± 0.00033	0.03101	0.19476 ± 0.00032	0.03070

In contrast to the full protein rmsd, the rmsds of separate domains show more scattering (more *σ*) for eEF1A2 than for eEF1A1. So, the internal motions of the domains are larger in the eEF1A2 molecule. The difference in mobility of the two protein variants is mostly observed for domain III [see Additional file 3]. One may conclude that domain III of eEF1A2 moves in solution more away from the initial conformation than the same domain of eEF1A1. Thus, for eEF1A1 an increased mobility of the entire protein (inter-domain mobility) while for eEF1A2 an elevated mobility of individual domains (internal domain mobility) are detected.

**Figure 3:**
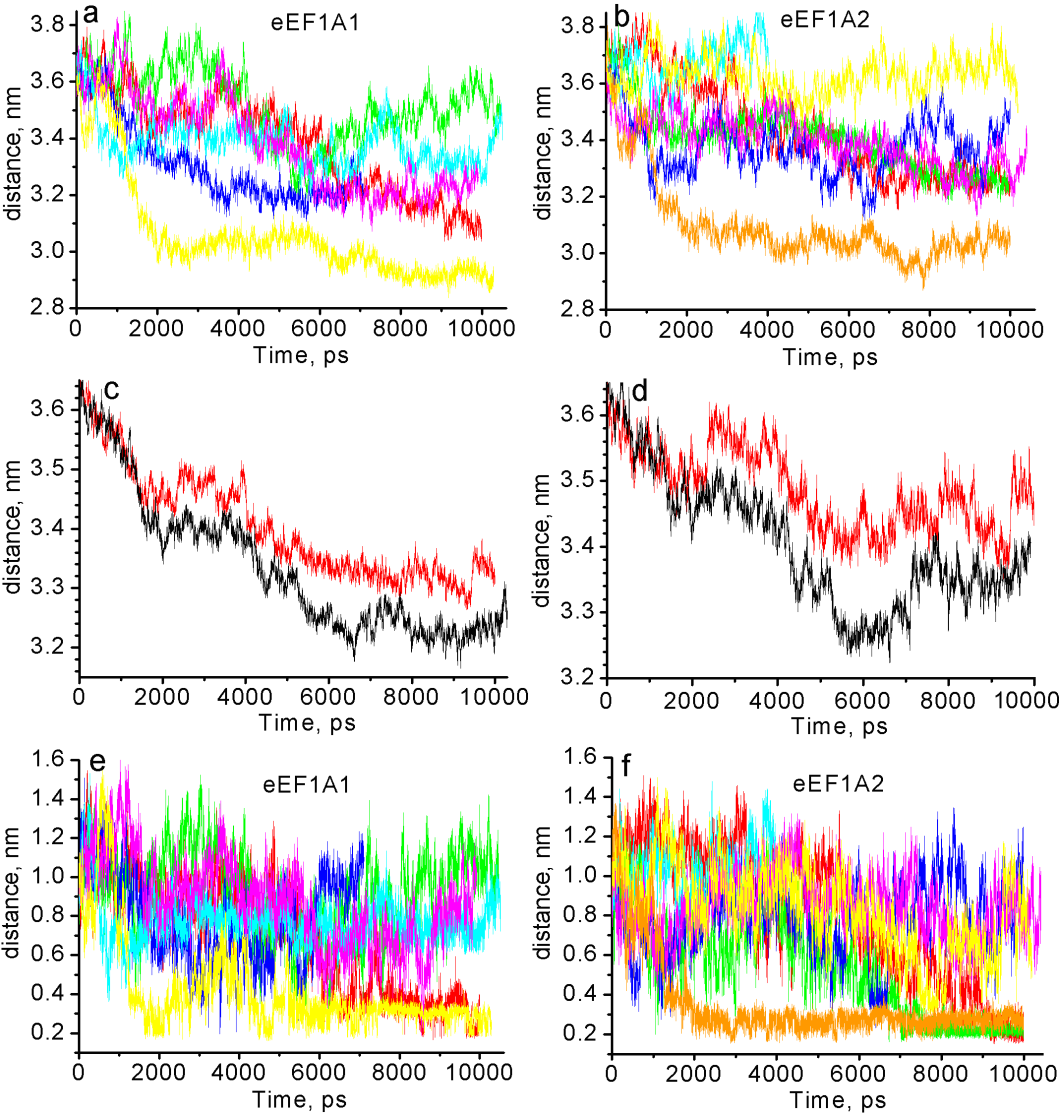
**Distance between centers of domains I and II (a-d) and minimal distance between regions Arg69-Leu77 and His295-Gly305 (e, f)**. a, e – eEF1A1: red – trajectory 1, green – trajectory 2, blue – trajectory 3, cyan – trajectory 4, magenta – trajectory 5, yellow – trajectory 6. b, f – eEF1A2: red – trajectory 7, green – trajectory 8, blue – trajectory 9, cyan – trajectory 10, magenta – trajectory 11, yellow – trajectory 12, orange – trajectory 13. c – the mean distances between centers of the domains I and II for eEF1A1 (black) and eEF1A2 (red); averaging has been done for all respective trajectories. d – the mean distances between the centers of domains I and II after the exemption of the trajectories characterized by the existence of "closed" conformation for more than 500 ps (1, 6 for eEF1A1 and 7, 8, 13 for eEF1A2).

The distance between centers of the domains was calculated (Figure 3) to directly analyze the inter-domain mobility of the isoforms. A direct link between the full protein rmsd and the distance between the domains I and II is observed for eEF1A1. The trajectories characterized by the maximal rmsd for the full protein (6 and 1) [see Additional file 3] demonstrate the smallest distance between domains I and II (Figure 3a). Similarly, the trajectories with the minimal rmsd (2 and 4) show the maximal distance between the domains I and II. Consequently, the departure of eEF1A1 from the initial conformation is accompanied by the approaching of domains I and II.

The correlation for eEF1A2 is less obvious. Thus, trajectory 13 demonstrating the maximal full protein rmsd is characterized by the minimal distance between the domains I and II (Figure 3b), but trajectory 12 which displays the elevated full protein rmsd is characterized by the largest distance between domains I and II. So, for both variants the correspondence between the maximal rmsd and the minimal distance between the domains I and II is conserved, while the correlation of the minimal rmsd and the maximal distance between the domains is valid only for eEF1A1. This confirms that the full protein rmsd scattering for eEF1A1 is determined mainly by the reciprocal motions of the domains I and II, whereas the rmsd scattering for eEF1A2 is determined both by the inter-domain and internal domain motions.

The average distance between domains I and II is less for eEF1A1 (Figure 3c and Table 1). The distance between the domains is decreased with simulation time for all trajectories of eEF1A1 (Figure 3a), while for eEF1A2 the abatement is less substantial and is not observed for trajectories 10 and 12 (Figure 3b).

The distances between domains I and III and between domains II and III change less significantly than distance between domains I and II for both isoforms. However, increased scattering (higher *σ*) of the distance between domains I and III for eEF1A1 as compared to eEF1A2 should be noted (Table 1).

Interestingly, eEF1A1 has more inter-domain mobility but less average distance between domains I and II, while eEF1A2 is characterized by lower inter-domain mobility but a larger average distance between the first and second domains. For both protein variants the average protein conformation is characterized by less distance between domains I and II as compared to the initial model. In the course of simulation the distance between the domains decreases frequently and increases only rarely. That is why the lower average distance between domains I and II might be characteristic of a protein with higher inter-domain mobility.

The increased proximity of the domains I and II causes a decrease of the gap between the domains, i.e. between chains Arg69-Leu77 of the domain I and His295-Gly305 of domain II (see Figure 2a). The minimal distance between the chains decreases up to van der Waals radii sum for trajectories 1 and 6 [see Additional file 4] of eEF1A1 (Figure 3e) and for trajectories 7, 8, 13 [see Additional file 5] of eEF1A2 (Figure 3f) indicating the formation of a "completely closed" conformation. This conformation is characterized by a high full protein rmsd with respect to the initial protein conformation and by a large number of contacts between the Arg69-Leu77 and His295-Gly305 groups.

The proteins can oscillate from an extended to a compact conformation and vice versa. For example, trajectory 6 moves to a "closed" conformation of a protein at 1300 ps, then returns to an "open" conformation at 2900 ps, and again transforms into a "closed" one at 4300 ps (Figure 3e,g). Trajectories 5, 9 and 12 adopt a "closed" conformation of a protein only for a short time at 8500–8600, 6350–6650 and 7500–7900 ps, respectively (Figure 3e–h). Critically, it has been demonstrated recently by small angle neutron scattering experiments that eEF1A1 adopts an "extended" conformation in solution, becoming more compact in the presence of tRNA [17]. Thus, one may suggest that the short-lived "closed" conformation of eEF1A found by MD simulation analysis can be stabilized by its biological ligands.

Importantly, the distance between the domains I and II remains closer for eEF1A1 than for eEF1A2 even after omitting from the calculations the trajectories which adopt a "closed" conformation for more than 500 ps (1, 6 for eEF1A1 and 7, 8, 13 for eEF1A2) (Figure 3d). Thus, the average distance between domains I and II is less in the eEF1A1 than in eEF1A2 "open" conformations. Since the region of the cleft between the domains I and II could be important for tRNA binding, at least for prokaryotic EF-Tu [18,19], it is reasonable to assume that the tRNA affinity for eEF1A1 and eEF1A2 could differ.

To describe the correlated motions of the protein domains, a covariance analysis of C_*α*_-atoms was performed (see Methods). Trajectories 2 [see Additional file 6], 4 and 5 for eEF1A1 and 9 [see Additional file 7], 11 and 12 for eEF1A2 were chosen as the most stable trajectories by C_*α*_-atoms rmsd matrices. It was determined that the ranges 2500–10466, 5440–10514, 3960–9920, 1070–10000, 3780–10417 and 1130–10197 ps chosen for trajectories 2, 4, 5, 9, 11 and 12 respectively, contain protein conformations with minimal rmsds with respect to each other. The eigenvalues and cosine contents for projections of the trajectories onto first eight eigenvectors are listed in Table 2. Table 2 shows that the principal components are well defined for the trajectory 2 of eEF1A1. For other trajectories and especially for the trajectory 11 of eEF1A2 the cosine content for the eigenvector 1 is relatively high, so the estimations of the correlated motions for these trajectories are more approximate. In fact, this may provide further evidence that eEF1A2 has more intra-domain non-correlated diffusive motions than eEF1A1.

**Table 2:** First eight eigenvalues and cosine contents for the trajectories 2, 4, 5, 9, 11 and 12

Isoform	eEF1A1			eEF1A2		
Trajectory; time interval	2; 2500–10466 ps	4; 5440–10514 ps	5; 3960–9920 ps	9; 1070–10000 ps	11; 3780–10417 ps	12; 1130–10197 ps

Number of eigenvector	Eigenvalue					

1	2.92577	1.44506	2.00650	2.27184	2.30533	2.15311
2	1.68434	0.86893	1.36554	1.28574	0.78048	1.77254
3	0.68870	0.38034	0.47608	0.73461	0.46551	0.64839
4	0.46802	0.27333	0.29183	0.50336	0.29763	0.46346
5	0.32693	0.23964	0.26331	0.42775	0.27844	0.43969
6	0.28298	0.18535	0.22431	0.23191	0.20223	0.27086
7	0.18644	0.14662	0.15777	0.20346	0.18002	0.25996
8	0.16773	0.10737	0.13055	0.17295	0.16660	0.22564

	Cosine content					

1	0.03374	0.60652	0.63817	0.62554	0.89852	0.50174
2	0.02503	0.07350	0.52379	0.04251	0.22506	0.27044
3	0.00185	0.18572	0.25656	0.06206	0.00093	0.15005
4	0.00111	0.04537	0.30264	0.07576	3.209·10^-7^	0.02686
5	0.02844	0.00041	0.23341	0.19980	0.116878	0.00641
6	0.02734	0.03769	0.12111	0.07724	0.00179	0.10954
7	0.11791	0.00358	0.02747	0.02094	0.11159	0.02160
8	0.02168	0.16876	0.01118	0.08322	0.00296	0.11837

**Figure 4:**
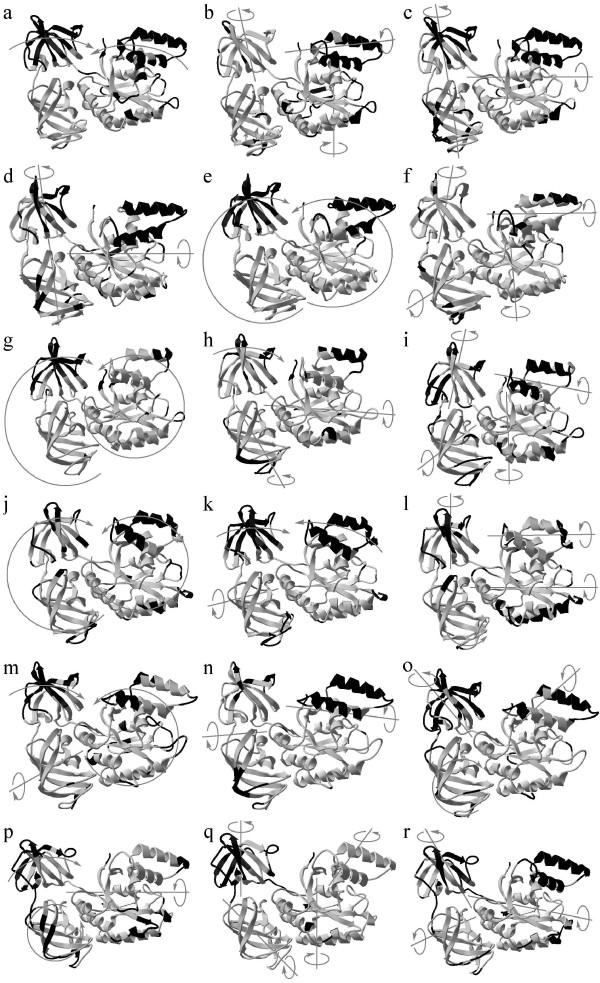
**Main correlated motions of two isoforms of human translation elongation factor 1A**. a-i – eEF1A1, j-r – eEF1A2. a-c – 2500–10466 ps of trajectory 2; d-f – 5440–10514 ps of trajectory 4; g-i – 3960–9920 of trajectory 5; j-l – 1070–10000 ps of trajectory 9; m-o – 3780–10417 ps of trajectory 11; p-r – 1130–10197 ps of trajectory 12. a, d, g, j, m, p – first eigenvector; b, e, h, k, n, q – second eigenvector; c, f, i, l, o, r – third eigenvector. The protein regions of the maximal C_*α*_-atom displacements (> 0.05 nm) are colored in black. The motions are shown on the average protein conformations in the respective trajectory ranges.

As can be seen from Figure 4, the main correlated motions for both isoforms are characterized by an increased proximity of the domains I and II (first principal components of trajectories 2, 5, 9, 11 and 12 and second principal components of trajectories 5, 9 and 11, Figure 4a,e,g,h,j,k,m,p) and by rotation of the structural domains around diverse axes. The domain II and "top" part of domain I (tail Met1-Lys5, helices M3l/Lys36-Glu48, Ala57-Glu68 and loops Cys31-Asp32, Met49-Tyr56, Arg69-Leu77) are most movable. In a number of cases "top" part of the domain I moves independently from the remaining part of the domain I (Figure 4a,b,f,i,k,l,n,o,q). In some cases the domains II and III rotate as a single whole around the same axis (Figure 4c,d,e,g,j,n).

Thus, we conclude that the main correlated motions of the two eEF1A isoforms are similar: the coming together of the domain II and the "top" part of domain I as well as rotation of structural domains.

**Figure 5:**
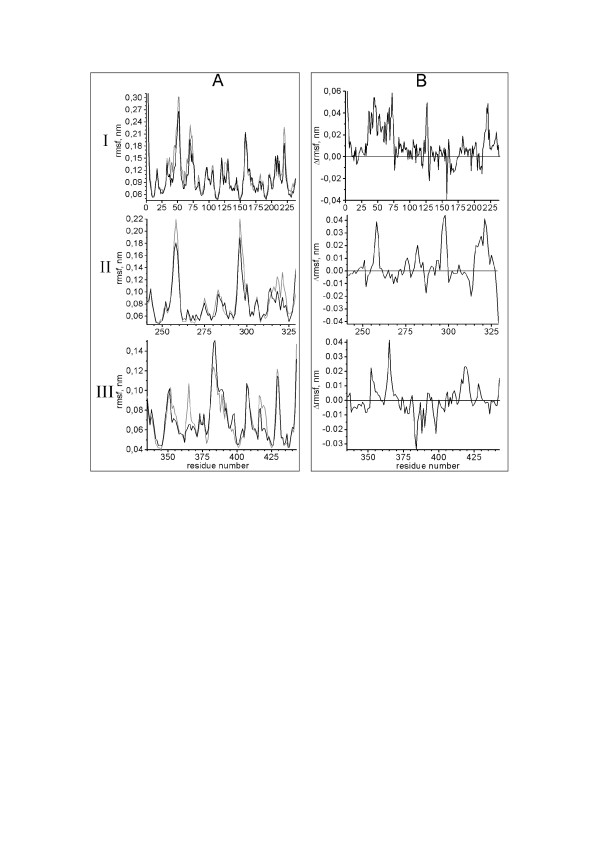
**Root-mean-square fluctuations of C-alpha atoms of the two eEF1A isoforms**. Data are averaged for the trajectories 1–6 of eEF1A1 and 7–9, 11–13 of eEF1A2. A – rmsf of eEF1A1 (black) and eEF1A2 (gray). B – difference between rmsf of eEF1A2 and eEF1A1. I – domain I, II – domain II, III – domain III.

To characterize the random, non-correlated motions of C_*α*_-atoms, the rmsf of these atoms were calculated for the separate domains in the 6000 ps trajectory ranges: 4000–10000 ps for trajectories 1, 2, 4, 6, 7, 8, 9, 11, 12 and 13, 1110–7110 ps for trajectory 3 and 3920–9920 ps for trajectory 5. The data were averaged for each variant (Figure 5). Figure 5 a demonstrates that the maximal flexibility of C_*α*_-atoms is typical for the loops, the "top" part of the domain I and the "bottom" of domain III (Pro350-Gln352, Arg381-Gly/Asn390). The residues for which a difference between the rmsf of eEF1A2 and eEF1A1 is more than 0.02 nm are labeled in red in Figure 2B, while residues with the negative difference less than -0.02 nm are labeled in blue.

### Calmodulin binding

**Figure 6:**
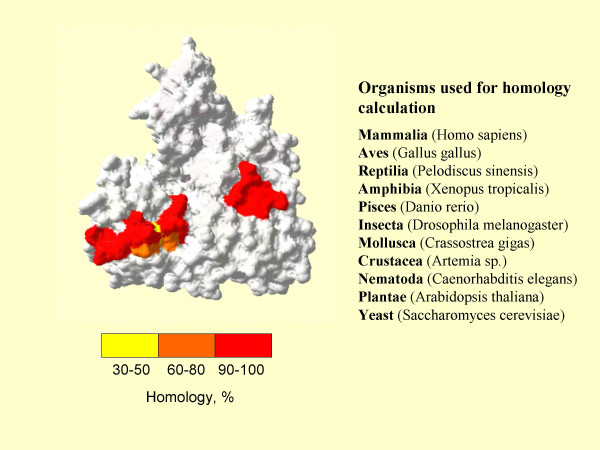
Conservation of exposed residues in putative calmodulin binding domain of the eEF1A homologues.

The importance of the data obtained for providing an explanation of the possible functional dissimilarity of the isoforms can be demonstrated by analysis of the putative calmodulin binding site. It is known that plant and Tetrahymena eEF1A are calmodulin-binding proteins [20,21]. Amino acid residues Asn311-Gly327, Gly422-Val437 in eEF1A1 and Arg427-Val437 in eEF1A2 (Figure 1a) are predicted by the Calmodulin Target Database (CTD) program [22] to be a possible calmodulin-binding site of human eEF1A. The amino acid residues comprising putative calmodulin binding site are well conserved from human to yeast (Figure 6), this finding is in line with functional importance of the site.

The Asn311-Gly327 motif is situated in domain II and contains an unfolded loop Lys313-Gly323 flanked by parts of neighboring *β*-strands. Because the Val325-Gly327 region has no solvent accessible surface, only residues Asn311-Asn324 could participate in calmodulin binding if no essential conformational changes of domain II take place during protein-protein interaction.

It was recently suggested that calmodulin-binding motifs in a protein should be disordered or flanked by disordered regions, adopting the most appropriate conformation for interaction with calmodulin [23]. Because region Lys313-Gly323 is disordered, the motif Asn311-Gly327 satisfies the criterion for calmodulin-binding targets [23].

**Figure 7:**
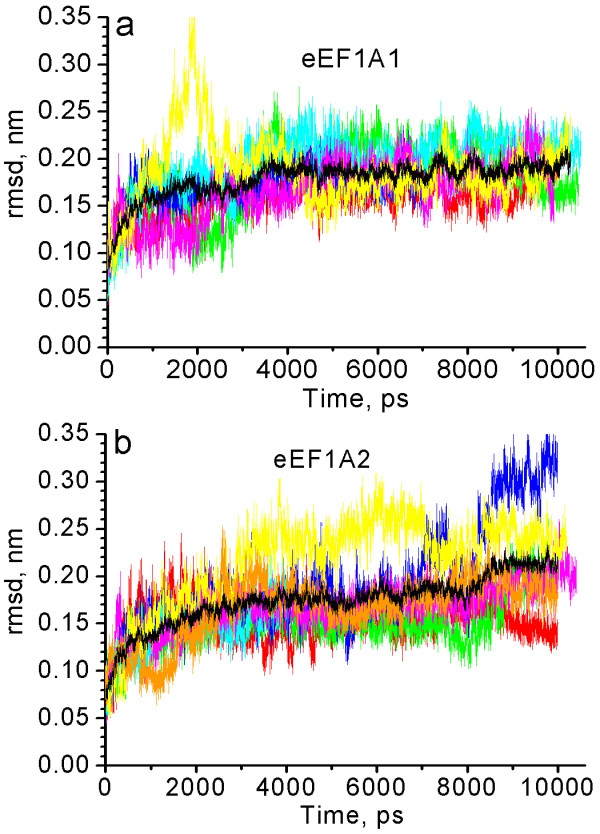
**C_*α*_-atoms rmsd of Asn311-Gly327 after fitting of domains II to the initial domain conformation**. a – eEF1A1: red – trajectory 1, green – trajectory 2, blue – trajectory 3, cyan – trajectory 4, magenta – trajectory 5, yellow – trajectory 6. b – eEF1A2: red – trajectory 7, green – trajectory 8, blue – trajectory 9, cyan – trajectory 10, magenta – trajectory 11, yellow – trajectory 12, orange – trajectory 13. Black – average curves.

On the other hand, the binding process is most favorable by entropy when the protein loses a minimal number of degrees of freedom, i.e. when the binding motif has minimal diffusive non-correlated motions before the binding. Thus, ordered regions such as *α*-helices flanked by disordered regions reveal an especially high inclination for calmodulin binding [23]. Figure 5b II shows that residues Val315-Val325 are more flexible in eEF1A2 than in eEF1A1 (with a difference between the rmsf of eEF1A2 and eEF1A1 of more than 0.01). Besides, the C_*α*_-atoms rmsds of the motif Asn311-Gly327 during the fitting of domain II to the initial conformation are more scattered in eEF1A2 than in eEF1A1 (Figure 7a,b and Table 1), so the diffusive motions of the motif residues are larger in the second isoform. Thus, the region Asn311-Gly327 could bind calmodulin more tightly in eEF1A1 than in eEF1A2.

As for the second putative calmodulin-binding motif, the region of Gly422-Val437 is situated on the interface between domains I and III and comprises a *β*-hairpin. The region is essentially buried and only residues Arg427-Gln431 have enough surface area accessible for interaction with other proteins. Therefore we believe the motif Gly422-Val437 has limited ligand binding capacity which, however, can be increased in the case of essential changes in the mutual orientation of domains I and III. Because inter-domain mobility is larger in eEF1A1 (Table 1), the reorientation of such domains is more likely to happen in that isoform. Furthermore, less significant diffusive mobility of Asp428 in eEF1A1 (Figure 5B III) should favor higher calmodulin-binding ability of that isoform.

Thus, the MD simulation analysis predicts that eEF1A1 isoform should have increased affinity for calmodulin.

**Figure 8:**
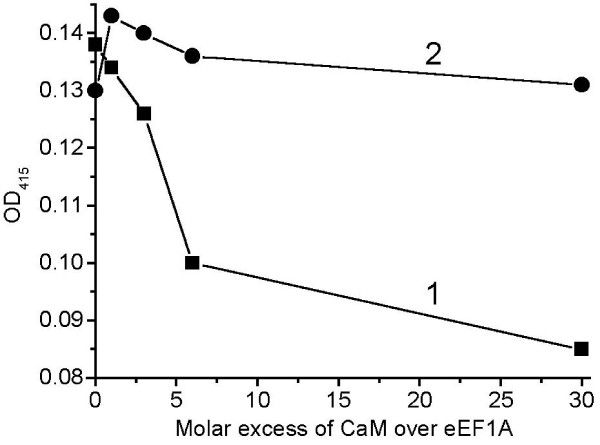
**Comparison of Ca^2+^-calmodulin binding to eEF1A isoforms by enzyme-linked immunosorbent assay-based binding assay**. 1 – eEF1A1, 2 – eEF1A2. Microtiter 96-well plates (Dynatech microtiter) were coated with purified eEF1A1 or eEF1A2, and monoclonal anti-eEF1A antibody was added with or without increasing amounts of Ca^2+^-calmodulin.

The ability of eEF1A1 and eEF1A2 to bind calmodulin was examined experimentally. An enzyme-linked immunosorbent assay-based binding assay was used to compare calmodulin-binding properties of the isoforms. eEF1A1 or eEF1A2 were pre-absorbed in the wells of a microtiter plate and Ca^2+^-calmodulin was added to compete with anti-eEF1A antibodies. If calmodulin binding to eEF1A challenges antibody binding the absorbance value is decreased in the presence of calmodulin. Ca^2+^-calmodulin was found to interact with the eEF1A1 isoform only (Figure 8), thus validating the MD simulation prediction. The binding of Ca^2+^-calmodulin to the eEF1A1 isoform was concentration dependent and observed at a 6-fold molar excess of calmodulin over eEF1A1. Importantly, no Ca^2+^-calmodulin interaction with eEF1A2 was detected even at 30-fold excess of the ligand. Anti-eEF1A antibodies did not show any affinity for Ca^2+^-calmodulin. The addition of Ca^2+ ^alone (in the absence of calmodulin) to compete with anti-eEF1A antibodies did not interfere with the absorbance.

## Conclusion

The dynamic behavior of eEF1A – one of the main protein components of the human translation machinery – has been described for the first time. The existence of a reversible transition between "open" and "close" conformations of eEF1A gives a molecular background for the demonstrated earlier ability of eEF1A to change shape upon interaction with tRNA.

We showed how a tiny divergence in the amino acid sequences of the protein variants possessing 98% similarity could still lead to changes of the spatial structure and dynamics of the isoforms.

Different affinity of the eEF1A1 and eEF1A2 isoforms for the important signaling protein calmodulin has been predicted by MD data and demonstrated experimentally. Thus, diverse dynamic structures of the isoforms could alter the types of interaction with signaling proteins. This finding gives important background information to consider in the context of specific cancer-related properties of eEF1A2 [7-9]. Experiments to inspect the MD results by a number of biophysical and molecular biology methods are now in progress.

## Methods

The three-dimensional models of the two variants of human eEF1A have been built by the Swiss-Model server [24,25] using the crystallographic structures of elongation factors 1A of yeast Saccharomyces cerevisiae [PDB: 1IJE, PDB: 1IJF, PDB: 1G7C, PDB: 1F60] and archaebacteria Sulfolobus solfataricus [PDB: 1JNY] as templates. The identity of yeast eEF1A and human eEF1A1 and eEF1A2 variants is 80.7 and 79.6% respectively (Figure 1), and the identity of archaebacterial and human eEF1A is 53.1% for eEF1A1 and 52.2% for eEF1A2. It should be noted that the only crystallographic structure of eukaryotic factor 1A available is that of yeast eEF1A in the complex with part of the eEF1Balpha molecule [11,12]. eEF1A was in nucleotide-free form in the complex. We do not know at present how the presence of a nucleotide would influence the molecular dynamics studies. Meanwhile, contrary to prokaryotic analogue EF-Tu, no marked difference between GDP and GTP-bound conformations of eEF1A was found [1]. Further studies are necessary to make more definite conclusion.

The C-termini of yeast and archaebacterial elongation factors 1A are unstructured, and that is why amino acid residues homologous to residues 444–462 of the mammalian eEF1A were not determined by X-ray diffraction [11,12,26]. So the residues were not included in resulting models.

Trimethylation of lysine residues 36, 79, 318 of eEF1A1 [10] and 55, 165 of eEF1A2 [5] was performed by replacing of hydrogen atoms by methyl groups. Similarly, Lys55 and Lys165 of eEF1A1 were changed to N-dimethyllysines. NH_2_-groups of Gln301 and Gln374 of both protein variants were replaced by glycerylphosphoryletanolamines [5,10].

The procedure of MD simulation was performed as in [27]. The simulation was done using the GROMACS 3.1.4 software package [28,29]. The GROMOS96 43a2 force field [30] was modified by the inclusion of parameters for N-methyllysine, N-dimethyllysine, N-trimethyllysine and L-glutamyl 5-glycerylphosphorylethanolamine [see Additional files 8 and 9]. The GROMOS96 topology data for the mentioned non-standard amino acid residues were generated by the Dundee PRODRG2 Server [31,32].

Hydrogens were added to the non-carbon heavy atoms using the pdb2gmx program of the GROMACS 3.1.4 package. The lysines, dimethyllysines, arginines and N-terminal amine group were put in the protonated state with charge +1. The carboxyl groups of aspartic and glutamic acids and of the C-terminal residue are deprotonated and charged negatively. The His7 residue of both variants is protonated at the N_*ε *_position since in the case of N_*δ *_protonation the hydrogen atom would be too close to CH_*δ *_of Tyr86. His15, 95 and 197 are protonated at N_*ε *_because the N_*δ *_atoms of the residues can form hydrogen bonds with hydrogens of NH-groups of Asp17 and Asp97 and with hydroxyl hydrogen of Ser194, respectively. His26, His296 and His367 are protonated at N_*δ*_, allowing the electrostatic interaction of the protons with carbonyl oxygens of the same residues. His136 is protonated at the N_*δ *_position that allows the N_*ε *_atom to form a H-bond with amide hydrogen of the Gln132 side chain. His295 of both eEF1A isoforms is protonated at *ε*-nitrogen, since that allows the proton to interact with the N_*ε *_atom of His296. His349 is protonated at the N_*δ *_position, because if N_*ε *_is protonated, it would be too close to Met429. His364 was determined to be a N_*δ*_-protonated residue since in that case the N_*δ *_hydrogen can interact with the sulfur atom of Cys363.

The protein models were inserted into the virtual boxes of a truncated octahedron shape by the editconf program. The minimal distance between the protein and the box wall was 1.5 nm to prevent artificial periodicity [33,34] and to allow the proteins to change conformations freely. The box volumes were 1253.76 and 1303.49 nm^3 ^for eEF1A1 and eEF1A2, respectively. The difference in the box volume sizes is explained by different orientation of the GPE side chains and by the presence of differently sized amino acid residues (Mly/M3l165, Ala/Pro206, Thr/Glu217, Asp/Glu220, M3l/Lys318) at the eEF1A1 and eEF1A2 surfaces. 39463 and 41270 SPC (Single Point Charge) [35] water molecules were added into the boxes containing the eEF1A1 and eEF1A2 variants respectively (genbox program). The 69 and 72 water molecules were replaced by sodium ions, whilst the 74 and 77 H_2_O molecules were replaced by chlorine ions for eEF1A1 and eEF1A2 correspondingly, to neutralize the system and to mimic the ionic force of 0.1 M (genion program). The positions of the ions were chosen by the Poisson-Boltzmann distribution. The energy minimization of the system was conducted by alternating steepest descent and conjugative gradient algorithms up to the energy gradient less than 100 kJ/(mol·nm). The solvent molecules equilibration was performed by the 500 ps MD simulation with the protein atoms restrained to their positions. The initial atom velocities were generated from the Maxwell's distribution. Atoms coordinates were updated each 2·10^-15 ^s. The protein bonds were constrained by the linear constraint solver (LINCS) algorithm [36]. The cut-off for electrostatic interaction was 0.9 nm. A double cut-off was used for the Lenard-Jones interaction treatment. The interactions between atoms within 0.9 nm were updated at each step, and the interactions within the distance between 0.9 and 1.4 nm were updated at each 10^th ^step. The Particle-mesh Ewald (PME) algorithm [37] was applied to describe the long-range electrostatic interactions. Temperature and pressure were kept at 298 K and 1 atmosphere using the Berendsen's method [38] with relaxation times of 0.1 ps and 0.5 ps, respectively. After equilibration of solvent molecules, additional energy minimization of the system was carried out. Then, the main MD simulation was performed with the same parameters as the restrained simulation except the control of pressure. The atom coordinates were written into the output trajectory file every 1 ps.

Multiple MD simulation was performed. Six trajectories were obtained for the eEF1A1 isoform: 1 (10000 ps), 2 (10466 ps), 3 (7110 ps), 4 (10514 ps), 5 (9920 ps) and 6 (10297 ps), while seven trajectories were simulated for the eEF1A2 isoform: 7 (10000 ps), 8 (10000 ps), 9 (10000 ps), 10 (4015 ps), 11 (10417 ps), 12 (10197 ps), and 13 (10000 ps). The different initial velocities of atoms were set for the different trajectories. The trajectory analysis was performed using following parameters:

1) C_*α*_-atoms trace root-mean-square deviation (rmsd) after fitting to the initial conformation before the main dynamics (g_rms program). Since the protein domains can fluctuate relative to each other, the rmsd is calculated both for the whole protein and for the separate domains. To determine the flexibility of the putative calmodulin-binding motifs (Asn311-Gly327 in domain II and Gly422-Val437 in domain III) the fitting was done by C_*α*_-atoms of the respective domain and the rmsd was calculated for C_*α*_-atoms of the calmodulin-binding motif.

2) The distances between the centers of the domains (g_dist program). Centers of domains were calculated as mean values of coordinates of C_*α *_atoms of corresponding domains.

Parameters 1) and 2) calculated from MD trajectories after 4000 ps were averaged for each eEF1A isoform and deviations *σ *of the parameters were computed (Table 1). Besides, deviations *m *of average values were calculated:

$$m=\sigma /\sqrt{n},\;$$

where *n *is total number of frames in all trajectories after 4000 ps for one isoform; *n *= 34307 and 36629 for eEF1A1 and eEF1A2, respectively. The Student's coefficient for a 0.05 significance level was taken as 2.

3) The minimal distance between the residues Arg69-Leu77 and His295-Gly305 (g_mindist program).

4) The root-mean-square fluctuations (rmsf) of C_*α*_-atoms with respect to their average positions after fitting to the initial conformation (g_rmsf program). This value was calculated for the separate protein domains along trajectory ranges of 6000 ps.

5) The analysis of the correlated motions of C_*α*_-atoms (g_covar, g_anaeig and g_analyse programs).

Detailed examination of the correlated protein motions has been conducted by C_*α*_-atoms trace covariance analysis [39] of the most stable trajectories. First of all, the C_*α*_-atoms rmsd matrices *M *are built using the g_rms program. The matrices have a dimension n × n, where n is the number of the trajectory frames (equal to the number of picoseconds in the trajectory). Each matrix element *M*_*i*, *j *_is the rmsd between the protein conformations at time moments *i *and *j*. The trajectory ranges presenting the conformations characterized by the least rmsd with respect to each other have been determined. The covariance analysis was performed for these trajectory ranges and the covariance matrices *C *have been constructed:

$$C_{k,l}=\langle \vec{x_{k}(t)-\langle x_{k}(t)\rangle }\rangle \times \langle \vec{x_{l}(t)-\langle x_{l}(t)\rangle }\rangle ,$$

where the arrows above the expressions denote vector values, the angle brackets – average values, the × sign is the scalar product, *x *is the coordinate, *t *– time, *k *and *l *are one of the space dimensions (*x*, *y *or *z*) for one of the atoms. So the covariance matrices have 3 m × 3 m dimensions, where m is the total number of C_*α*_-atoms in the protein model. If the two atoms move along the two dimensions absolutely asynchronously, the matrix element is equal to zero. If they move absolutely synchronously, the matrix element corresponds to the atoms rmsf. As an atom moves synchronously with itself, the covariance matrix diagonal contains the corresponding atoms rmsf along certain dimensions. The matrix *C *has been diagonalized using orthonormal matrix *R*:

*C *= *R*·diag (*λ*_1_, *λ*_2_,..., *λ*_3*m*_)·*R*^*T*^,

where the columns of matrix *R *are eigenvectors, which correspond to eigenvalues *λ*, *λ*_1 _≥ *λ*_2 _≥ ... ≥ *λ*_3m_. The first few eigenvectors (characterized by the largest eigenvalues) often reflect collective global motions of the protein.

The quality of the covariance analysis must be controlled to exclude interpretation of the random diffusion of atoms as the correlated one. In the case of random diffusion, the projection of the trajectory on eigenvector *k *(called also the principal component) is the cosine with a period of *t*_∞_·*k*/2, where *t*_∞ _is the length of the analyzed part of the trajectory. That is why the cosine content of the trajectory projections is calculated using the g_analyse program. The cosine content is equal to 1 if the trajectory projection is completely cosinusoid with the respective period and the motions of atoms are completely random. If the atom motions along respective eigenvectors are completely correlated, the cosine content is zero. So the cosine content can be considered as a fraction of the atoms motion randomness.

The programs VMD [40] and Swiss-PDB Viewer [41] were used for the trajectory visualization and graphical analysis of the resulting conformations.

The eEF1A1 and eEF1A2 isoforms were isolated from rabbit liver and muscle respectively as described by us earlier [42]. The activity of the eEF1A proteins was tested in a GDP/[^3^H]GDP exchange test as in [43]. Calmodulin was isolated from bovine brain according to conventional procedure [44].

In vitro binding of eEF1A (eEF1A1 and eEF1A2) with Ca^2+^-calmodulin was measured by an indirect Enzyme-linked Immunosorbent Assay using the procedure described in [45]. Purified eEF1A*GDP (0,5 *μ*g) in 100 mkl of TS buffer (30 mM Tris-HCl, pH 7,5; 10 mM KCl) was coated overnight at 4°C in a 96-well polystyrene microtiter plate (Dynatech microtiter). The wells were rinsed five times with 200 *μ*l of TS buffer. After blocking with 200 *μ*l of blocking buffer (0.1% bovine serum albumin in TS) for 1 h at room temperature and washing five times with 200 *μ*l of TS, 100 *μ*l of 2000-fold-diluted in blocking buffer mouse monoclonal anti-eEF1A antibody and varying amounts of Ca^2+^-calmodulin (1, 5 *μ*g, 5 *μ*g, 10 *μ*g and 50 *μ*g) were added to wells and incubated at room temperature for 2 h. Ca^2+^-calmodulin was prepared by addition of CaCl_2 _to calmodulin (final concentration 1 mM) After washing five times with 200 *μ*l of TS buffer, 100 *μ*l of 5000-fold-diluted secondary goat anti-mouse IgG antibody conjugated to horseradish peroxidase were added per well and incubated for 1 h at room temperature. Then unbound antibodies were removed by five washes of 200 *μ*l of TS buffer. After addition of 100 *μ*l of ABTS (2,2'-azino-bis(3-ethylbenzthiazoline-6-sulphonic acid) at 0.5 mg/ml, the incubation proceeded at room temperature for 10–15 min. The absorbance was estimated at 405 nm in a Tecan Sunrise ELISA plate reader. The experiments were performed several times to confirm reproducibility.

## Authors' contributions

DSK carried out MD simulation, MD analysis and drafted the manuscript; OVN participated in MD data analysis and carried out the biochemical experiments; CMA participated in design of the study and helped to draft the manuscript, BSN developed the concept of the study and participated in its coordination, AVE participated in data analysis and interpretation and helped to draft the manuscript. All authors read and approved the final manuscript.

## Supplementary Material

Additional file 1 Spatial model of human eEF1A1 with posttranslational modifications, in PDB format. Click here for file

Additional file 2 Spatial model of human eEF1A2 with posttranslational modifications, in PDB format. Click here for file

Additional file 3 The C_*α*_-atoms trace root-mean-square deviation from the initial protein conformation. a, b – full protein, c, d – domain I, e, f – domain II, g, h – domain III. a, c, e, g – eEF1A1: red – trajectory 1, green – trajectory 2, blue – trajectory 3, cyan – trajectory 4, magenta – trajectory 5, yellow – trajectory 6. b, d, f, h – eEF1A2: red – trajectory 7, green – trajectory 8, blue – trajectory 9, cyan – trajectory 10, magenta – trajectory 11, yellow – trajectory 12, orange – trajectory 13. Black – average curves. Click here for file

Additional file 4 Movie of eEF1A1 molecular dynamics, trajectory 6, cartoon representation. "Closed" conformation is formed. Click here for file

Additional file 5 Movie of eEF1A2 molecular dynamics, trajectory 13, cartoon representation. "Closed" conformation is formed. Click here for file

Additional file 6 Movie of eEF1A1 molecular dynamics, trajectory 2, cartoon representation. "Open" conformation is maintained. Click here for file

Additional file 7 Movie of eEF1A2 molecular dynamics, trajectory 9, cartoon representation. "Open" conformation is maintained. Click here for file

Additional file 8 File in .txt format. Content of the file should be inserted to the end of ffg43a2.rtp file of GROMOS96 43a2 force field for supporting non-typical amino acid residues: N-methyllysine (MLZ), protonated N-methyllysine (MLZH), N-dimethyllysine (MLY), protonated N-dimethyllysine (MLYH), N-trimethyllysine (M3L) and L-glutamyl 5-glycerylphosphorylethanolamine (GPE). Click here for file

Additional file 9 File in text format. Content of the file should be inserted to the end of ffg43a2.hdb file of GROMOS96 43a2 force field for supporting non-typical amino acid residues: N-methyllysine (MLZ), protonated N-methyllysine (MLZH), N-dimethyllysine (MLY), protonated N-dimethyllysine (MLYH), N-trimethyllysine (M3L) and L-glutamyl 5-glycerylphosphorylethanolamine (GPE). Click here for file

## References

[B1] Negrutskii BS, El'skaya AV (1998). Eukaryotic translation elongation factor 1 alpha: structure, expression, functions, and possible role in aminoacyl-tRNA channeling. Prog Nucleic Acid Res Mol Biol.

[B2] Petrushenko ZM, Budkevich TV, Shalak VF, Negrutskii BS, El'skaya AV (2002). Novel complexes of mammalian translation elongation factor eEF1A.GDP with uncharged tRNA and aminoacyl-tRNA synthetase. Implications for tRNA channeling. Eur J Biochem.

[B3] Thornton S, Anand N, Purcell D, Lee J (2003). Not just for housekeeping: protein initiation and elongation factors in cell growth and tumorigenesis. J Mol Med.

[B4] Ejiri S (2002). Moonlighting functions of polypeptide elongation factor 1: from actin bundling to zinc finger protein R1-associated nuclear localization. Biosci Biotechnol Biochem.

[B5] Kahns S, Lund A, Kristensen P, Knudsen CR, Clark BF, Cavallius J, Merrick WC (1998). The elongation factor 1 A-2 isoform from rabbit: cloning of the cDNA and characterization of the protein. Nucleic Acids Res.

[B6] Lamberti A, Caraglia M, Longo O, Marra M, Abbruzzese A, Arcari P (2004). The translation elongation factor 1A in tumorigenesis, signal transduction and apoptosis: review article. Amino Acids.

[B7] Anand N, Murthy S, Amann G, Wernick M, Porter LA, Cukier IH, Collins C, Gray JW, Diebold J, Demetrick DJ, Lee JM (2002). Protein elongation factor EEF1A2 is a putative oncogene in ovarian cancer. Nat Genet.

[B8] Tomlinson VA, Newbery HJ, Wray NR, Jackson J, Larionov A, Miller WR, Dixon JM, Abbott CM (2005). Translation elongation factor eEF1A2 is a potential oncoprotein that is overexpressed in two-thirds of breast tumours. BMC Cancer.

[B9] Kulkarni G, Turbin DA, Amiri A, Jeganathan S, Andrade-Navarro MA, Wu TD, Huntsman DG, Lee JM (2007). Expression of protein elongation factor eEF1A2 predicts favorable outcome in breast cancer. Breast Cancer Res Treat.

[B10] Dever TE, Costello CE, Owens CL, Rosenberry TL, Merrick WC (1989). Location of seven post-translational modifications in rabbit elongation factor 1 alpha including dimethyllysine, trimethyllysine, and glycerylphosphorylethanolamine. J Biol Chem.

[B11] Andersen GR, Pedersen L, Valente L, Chatterjee I, Kinzy TG, Kjeldgaard M, Nyborg J (2000). Structural Basis for Nucleotide Exchange and Competition with tRNA in the Yeast Elongation Factor Complex Eef1A:Eef1Ba. Mol Cell.

[B12] Andersen GR, Valente L, Pedersen L, Kinzy TG, Nyborg J (2001). Crystal Structures of Nucleotide Exchange Intermediates in the Eef1A-Eef1Balpha Complex. Nat Struct Biol.

[B13] Louise-May S, Auffinger P, Westhof E (1996). Calculation of nucleic acid conformation. Curr Opin Struct Biol.

[B14] Auffinger P, Louise-May S, Westhof E (1995). Multiple molecular dynamics simulations of the anticodon loop of tRNAAsp in aqueous solution with counterions. J Am Chem Soc.

[B15] Auffinger P, Louise-May S, Westhof E (1996). Molecular dynamics simulations of the anticodon hairpin of tRNAAsp: structuring effects of C-H···O hydrogen bonds and of long-range hydration forces. J Am Chem Soc.

[B16] Vaiana AC, Westhof E, Auffinger P (2006). A molecular dynamics simulation study of an aminoglycoside/A-site RNA complex: conformational and hydration patterns. Biochimie.

[B17] Budkevich TV, Timchenko AA, Tiktopulo EI, Negrutskii BS, Shalak VF, Petrushenko ZM, Aksenov VL, Willumeit R, Kohlbrecher J, Serdyuk IN, El'skaya AV (2002). Extended conformation of mammalian translation elongation factor 1A in solution. Biochemistry.

[B18] Nissen P, Kjeldgaard M, Thirup S, Polekhina G, Reshetnikova L, Clark BF, Nyborg J (1995). Crystal structure of the ternary complex of Phe-tRNAPhe, EF-Tu, and a GTP analog. Science.

[B19] Nissen P, Thirup S, Kjeldgaard M, Nyborg J (1999). The crystal structure of Cys-tRNACys-EF-Tu-GDPNP reveals general and specific features in the ternary complex and in tRNA. Structure.

[B20] Moore RC, Durso NA, Cyr RJ (1998). Elongation factor-1alpha stabilizes microtubules in a calcium/calmodulin-dependent manner. Cell Motil Cytoskeleton.

[B21] Ueno H, Gonda K, Takeda T, Numata O (2003). Identification of elongation factor-1alpha as a Ca2+/calmodulin-binding protein in Tetrahymena cilia. Cell Motil Cytoskeleton.

[B22] The Calmodulin Target Database. http://calcium.uhnres.utoronto.ca/ctdb/flash.htm.

[B23] Radivojac P, Vucetic S, O'Connor TR, Uversky VN, Obradovic Z, Dunker AK (2006). Calmodulin signaling: analysis and prediction of a disorder-dependent molecular recognition. Proteins: Struct Funct Bioinf.

[B24] Schwede T, Kopp J, Guex N, Peitsch MC (2003). SWISS-MODEL: an automated protein homology-modeling server. Nucleic Acids Research.

[B25] Swiss-Model. An Automated Comparative Protein Modelling Server. http://swissmodel.expasy.org//SWISS-MODEL.html.

[B26] Vitagliano L, Masullo M, Sica F, Zagari A, Bocchini V (2001). The Crystal Structure of Sulfolobus Solfataricus Elongation Factor 1 Alpha in Complex with Gdp Reveals Novel Features in Nucleotide Binding and Exchange. EMBO J.

[B27] Kanibolotsky DS, Ivanova OS, Lisnyak VV (2006). Comparison of NMR and MD N-H bond order parameters: example of HIV-1 protease. Molecular Simulation.

[B28] Lindahl E, Hess B, van der Spoel D (2001). GROMACS. 3.0: a package for molecular simulation and trajectory analysis. J Mol Mod.

[B29] GROMACS: Fast, Free and Flexible MD. http://www.gromacs.org.

[B30] Schuler LD, Daura X, van Gunsteren WF (2001). An improved GROMOS96 force field for aliphatic hydrocarbons in the condensed phase. J Comput Chem.

[B31] Schuettelkopf AW, van Aalten DMF (2004). PRODRG – a tool for high-throughput crystallography of protein-ligand complexes. Acta Crystallographica D.

[B32] The Dundee PRODRG2 Server. http://davapc1.bioch.dundee.ac.uk/programs/prodrg/prodrg.html.

[B33] Hunenberger PH, McCammon JA (1999). Effect of artificial periodicity in simulations of biomolecules under Ewald boundary conditions: a continuum electrostatics study. Biophys Chem.

[B34] Weber W, Hunenberger PH, McCammon JA (2000). Molecular dynamics simulations of a polyalanine octapeptide under Ewald boundary conditions: influence of artificial periodicity on peptide conformation. J Phys Chem B.

[B35] Berendsen HJC, Postma JPM, van Gunsteren WF, Hermans J, Pullman B (1981). Interaction models for water in relation to protein hydration. Intermolecular Forces.

[B36] Hess B, Bekker H, Berendsen HJC, Fraaije JGEM (1997). LINCS: A linear constraint solver for molecular simulations. J Comp Chem.

[B37] Essman U, Perela L, Berkowitz ML, Darden T, Lee H, Pedersen LG (1995). A smooth particle mesh Ewald method. J Chem Phys.

[B38] Berendsen HJC, Postma JPM, DiNola A, Haak JR (1984). Molecular dynamics with coupling to an external bath. J Chem Phys.

[B39] Hess B (2002). Convergence of sampling in protein simulations. Phys Rev E.

[B40] Humphrey W, Dalke A, Schulten K (1996). VMD – Visual Molecular Dynamics. J Molec Graphics.

[B41] Guex N, Peitsch MC (1997). SWISS-MODEL and the Swiss-PdbViewer: An environment for comparative protein modeling. Electrophoresis.

[B42] Shalak VF, Budkevich TV, Negrutskii BS, El'skaya AV (1997). A fast and effective method for purification of elongation factor 1À from rabbit liver. Ukr Biokhim Zh.

[B43] Carvalho MD, Carvalho JF, Merrick WC (1984). Biological characterization of various forms of elongation factor 1 from rabbit reticulocytes. Arch Biochem Biophys.

[B44] Gopalakrishna R, Anderson WB (1982). Ca2+-induced hydrophobic site on calmodulin: Application for purification of calmodulin by phenyl-Sepharose affinity chromatography. Biochem Biophys Res Commun.

[B45] Anand M, Balar B, Ulloque R, Gross SR, Kinzy TG (2006). Domain and nucleotide dependence of the interaction between *Saccharomyces cerevisiae *translation elongation factors 3 and 1A. J Biol Chem.

[B46] Instantaneous computations of applied and scientific problems. High-performance computing cluster. http://www.cluster.kiev.ua/eng/?about.

[B47] Supercomputers of Institute of Cybernetics NAS of Ukraine. Rapid computations of any complexity tasks. https://cluster.icyb.kiev.ua.

