# Supplementary figures and images for: Multiple molecular dynamics simulation of the isoforms of human translation elongation factor 1A reveals reversible fluctuations between "open" and "closed" conformations and suggests specific for eEF1A1 affinity for Ca2+-calmodulin
Source: BMC Struct Biol. 2008 Jan 25;8:4. doi: 10.1186/1472-6807-8-4 (PMC2275276; doi:10.1186/1472-6807-8-4)

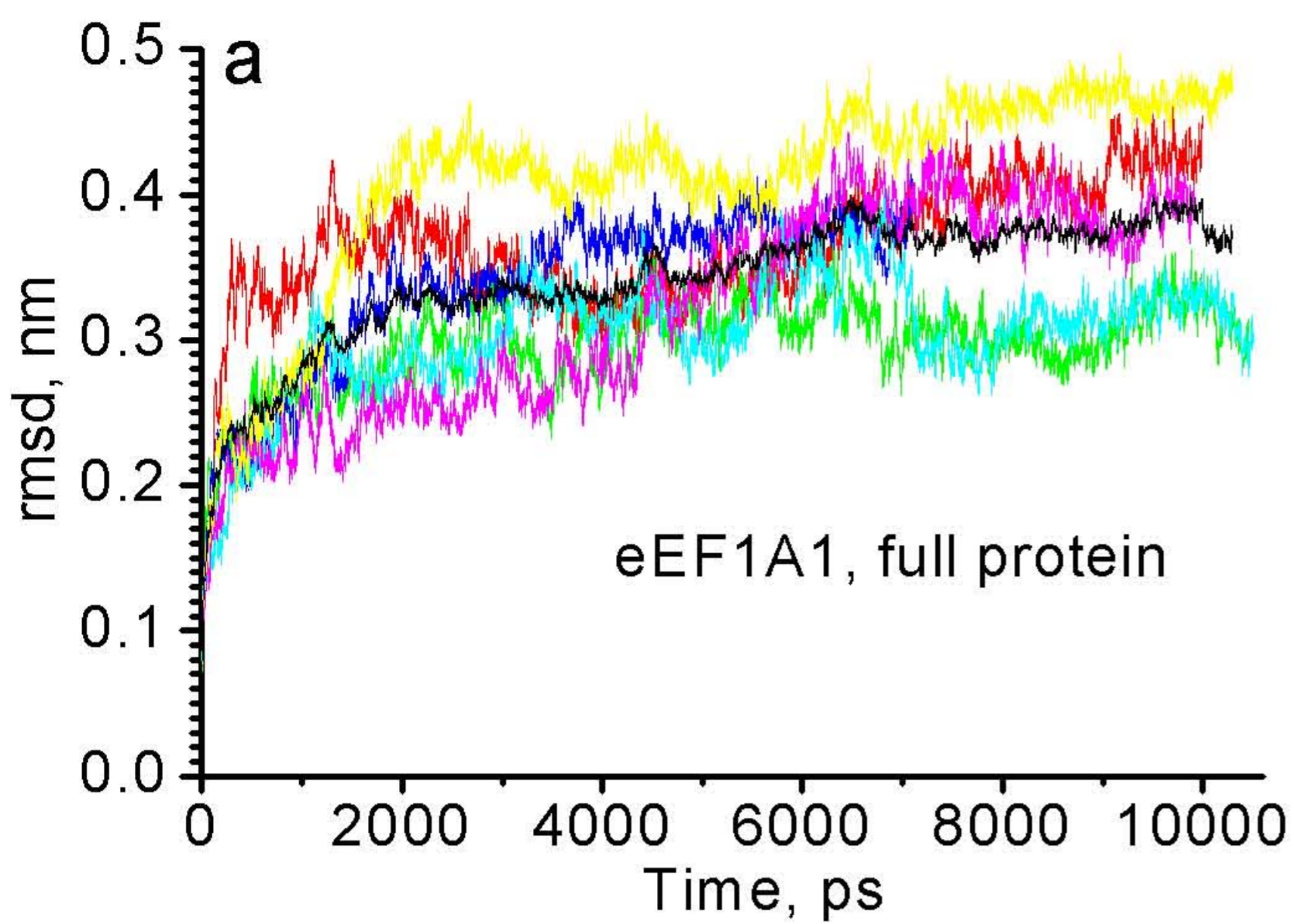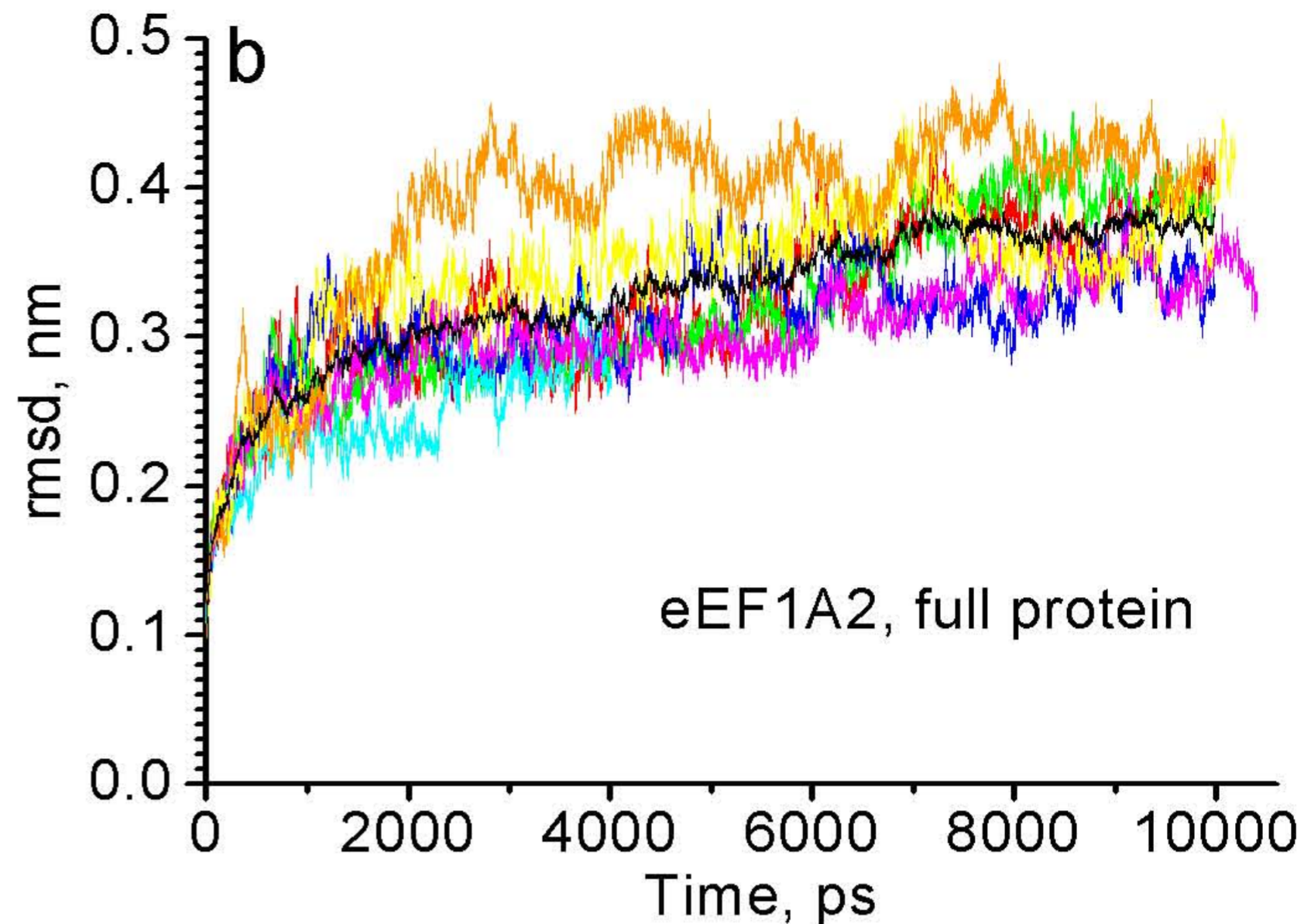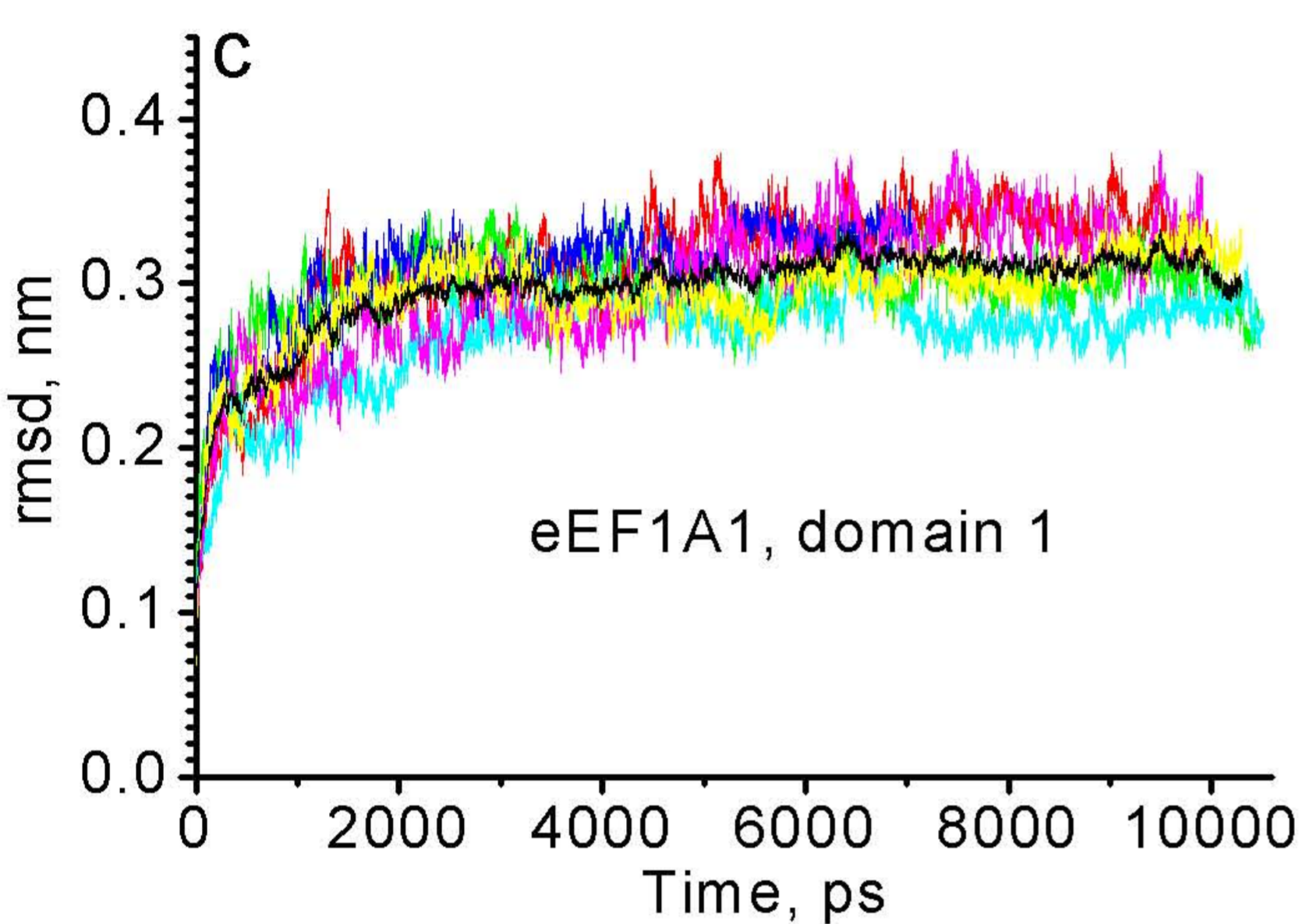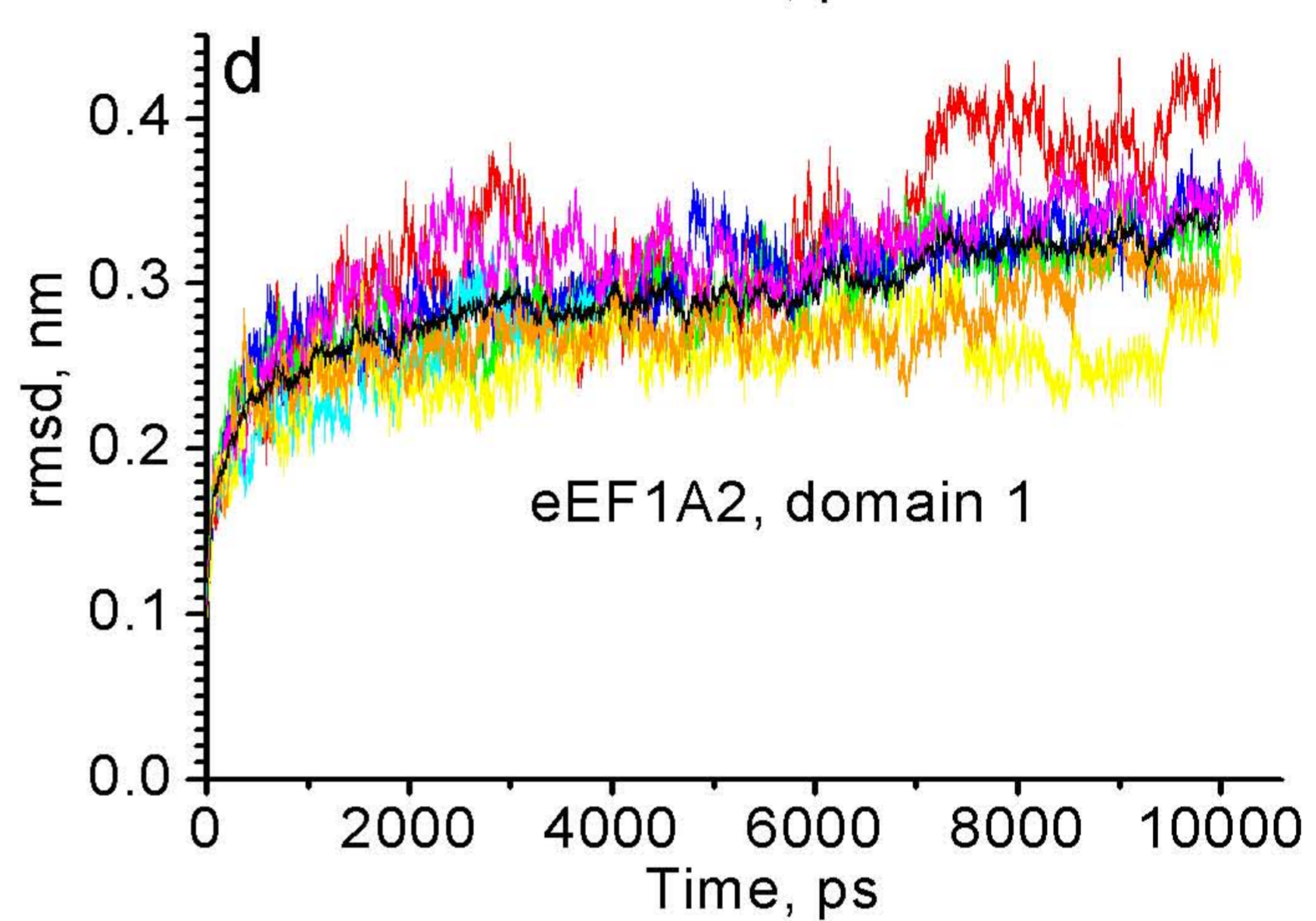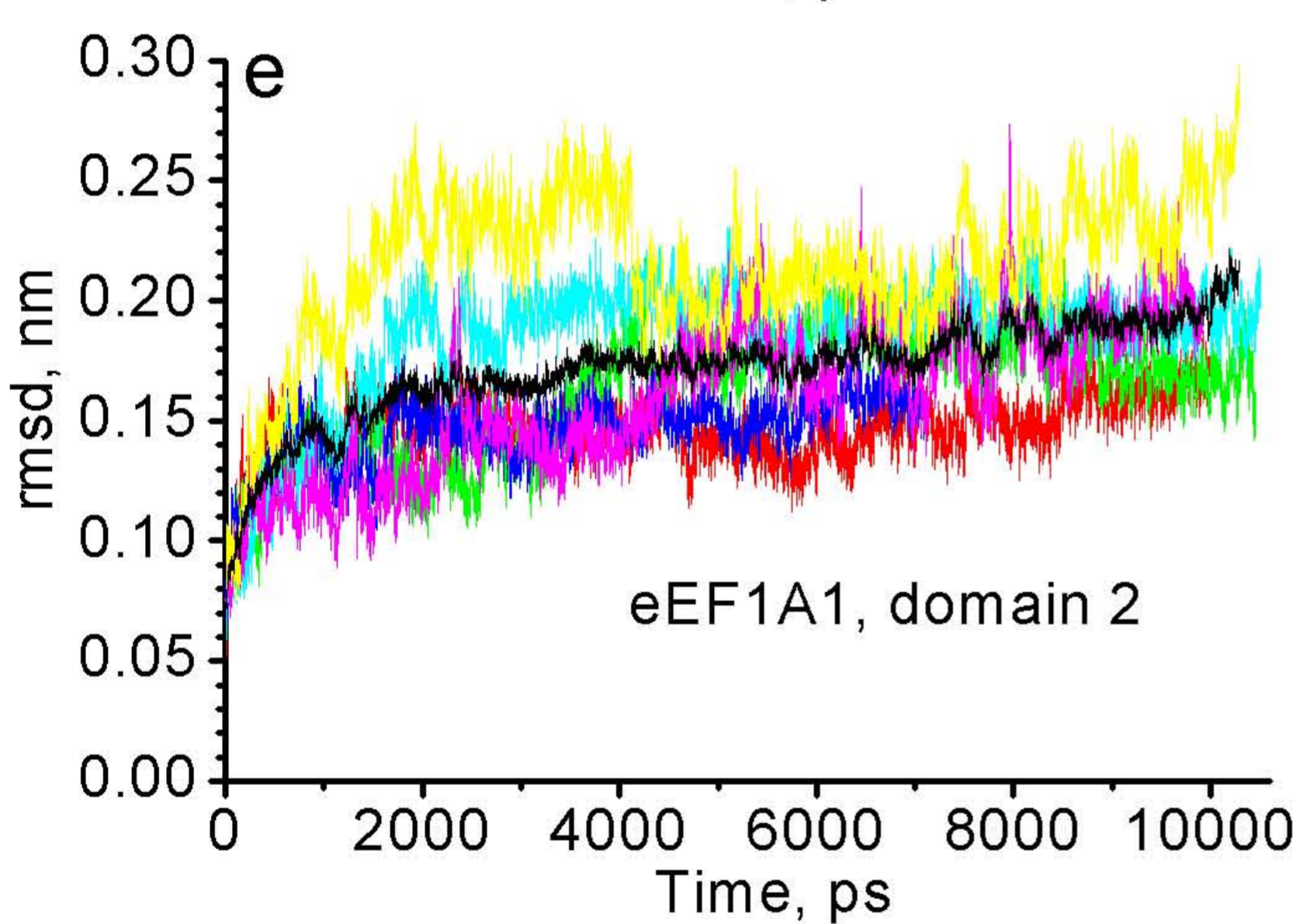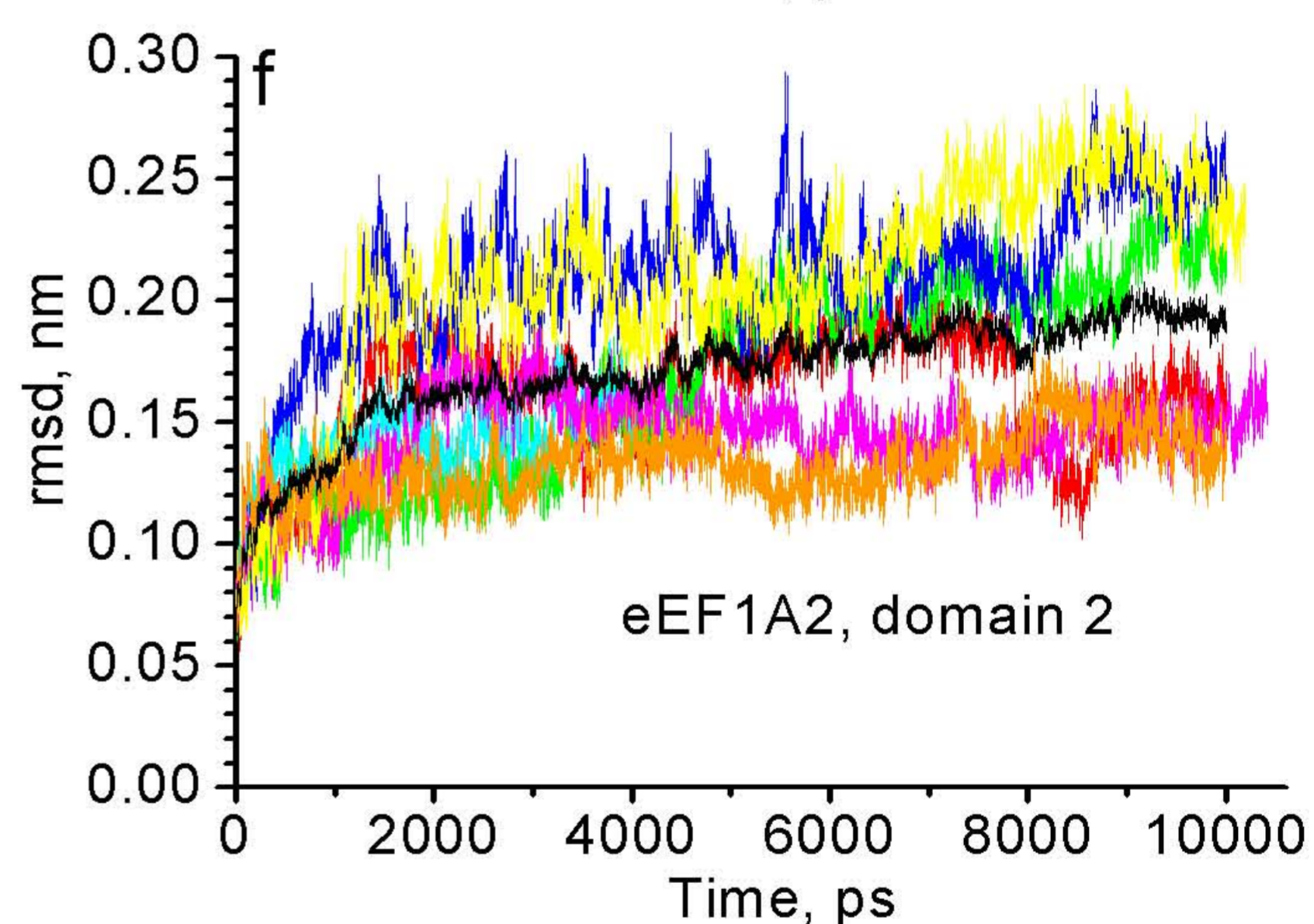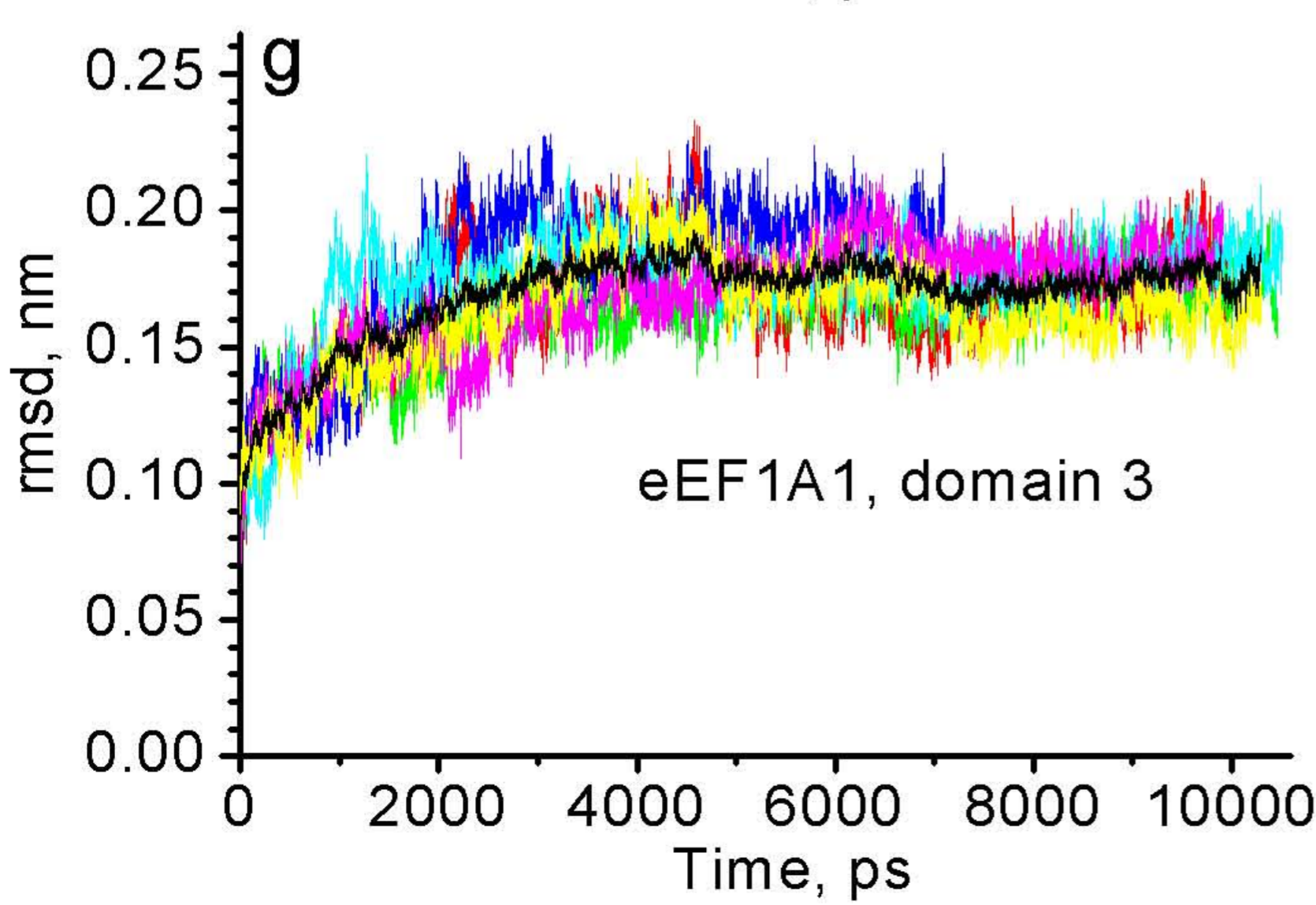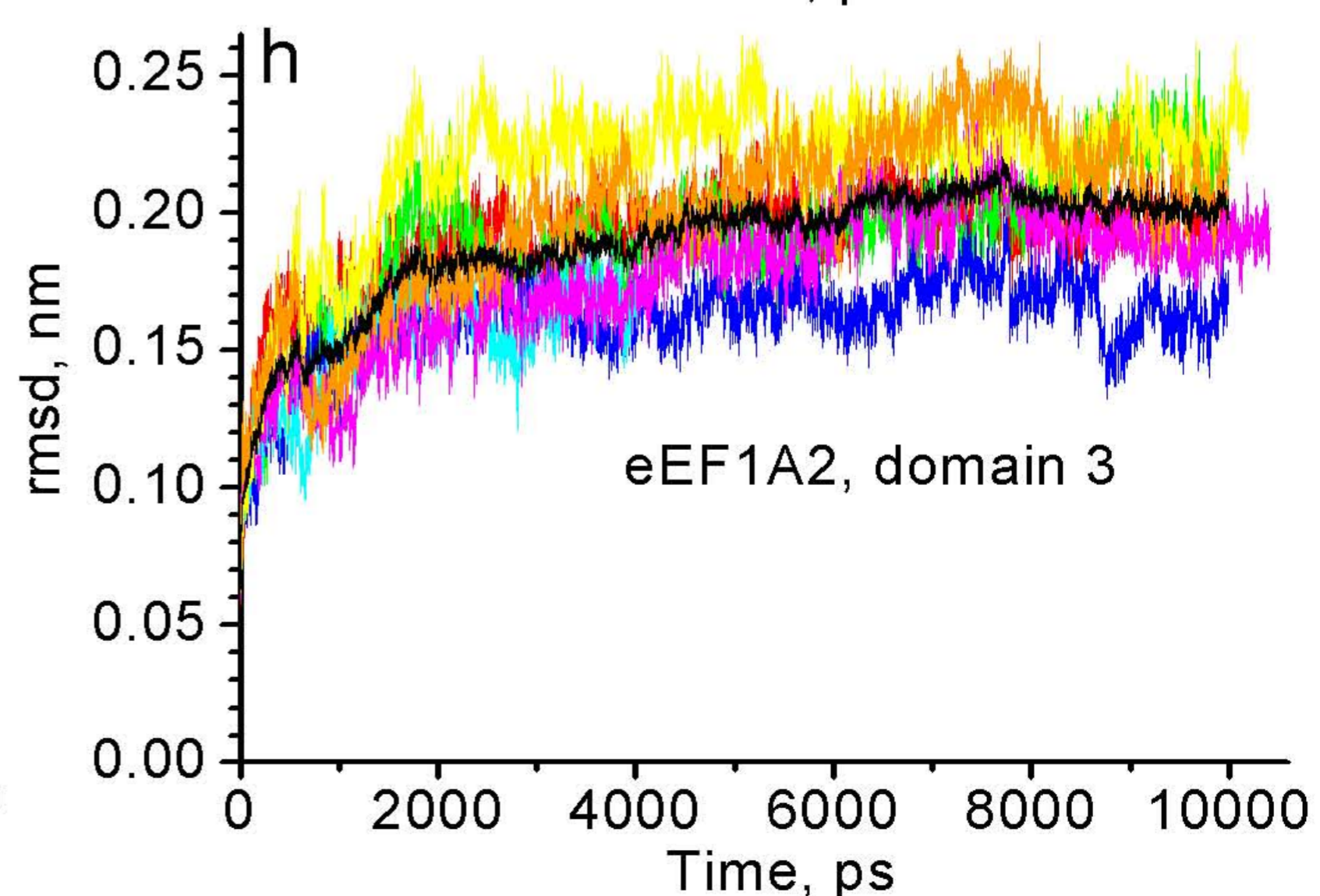

Supplement: Additional file 3 — The Cα-atoms trace root-mean-square deviation from the initial protein conformation. a, b – full protein, c, d – domain I, e, f – domain II, g, h – domain III. a, c, e, g – eEF1A1: red – trajectory 1, green – trajectory 2, blue – trajectory 3, cyan – trajectory 4, magenta – trajectory 5, yellow – trajectory 6. b, d, f, h – eEF1A2: red – trajectory 7, green – trajectory 8, blue – trajectory 9, cyan – trajectory 10, magenta – trajectory 11, yellow – trajectory 12, orange – trajectory 13. Black – average curves. [file 1472-6807-8-4-S3.pdf]
